# Evaluation of Symptomatic Improvement Following Functional Endoscopic Sinus Surgery in Chronic Rhinosinusitis: A Prospective Study

**DOI:** 10.7759/cureus.111454

**Published:** 2026-06-24

**Authors:** Riddhi Parmar, Hiren Doshi, Krunal Pandya, Radhika Parmar

**Affiliations:** 1 Otolaryngology, Shantabaa Medical College and General Hospital, Amreli, IND; 2 Otolaryngology, Narendra Modi Medical College, Ahmedabad, IND; 3 Pharmacology, Pandit Dindayal Upadhyay Medical College, Rajkot, IND

**Keywords:** anosmia, chronic rhinosinusitis, functional endoscopic sinus surgery (fess), nasal obstruction, symptom severity

## Abstract

Background

Chronic rhinosinusitis (CRS) is a prevalent inflammatory condition of the nasal cavity and paranasal sinuses characterized by symptoms persisting for more than 12 weeks, resulting in substantial impairment of quality of life. Functional endoscopic sinus surgery (FESS) is the established surgical treatment for patients who fail to respond to conservative medical management.

Methods

This prospective analytical study included 50 patients with medically refractory CRS who underwent FESS between April 2025 and March 2026. Four principal symptoms - nasal obstruction, facial pain, post-nasal drip, and anosmia - were scored preoperatively and at six months postoperatively using a four-point ordinal symptom severity scale (0 = absent, 1 = mild, 2 = moderate, 3 = severe). The Wilcoxon signed-rank test was used to compare preoperative and postoperative severity scores, with effect size calculated as r = Z/√N.

Results

The cohort comprised 32 males (64%) and 18 females (36%), with a mean age of 37.66 ± 13.95 years. Preoperative symptom prevalence was as follows: nasal obstruction in 92%, facial pain in 86%, anosmia in 74%, and post-nasal drip in 68%. Statistically significant reductions in mean severity scores were observed for nasal obstruction (2.40 to 1.66; p < 0.001, r = 0.99), facial pain (2.28 to 1.54; p < 0.001, r = 1.00), and post-nasal drip (1.54 to 1.24; p < 0.001, r = 1.00). Anosmia showed a non-significant trend toward improvement (1.58 to 1.44; p = 0.071, r = 0.47), with four patients demonstrating postoperative worsening. No patient experienced worsening of nasal obstruction, facial pain, or post-nasal drip. The complication rate was low, with minor bleeding in four (8%) patients and synechiae in three (6%) patients; no major complications were recorded.

Conclusions

FESS produced statistically significant and clinically meaningful improvements in nasal obstruction, facial pain, and post-nasal drip in patients with medically refractory CRS. Anosmia did not reach statistical significance, and its management in CRS warrants further investigation. The procedure was associated with a low complication rate, supporting its role as a safe and effective surgical intervention.

## Introduction

Chronic rhinosinusitis (CRS) is among the most common conditions encountered in otorhinolaryngology practice, with an estimated global prevalence of 10-12% in the adult population and a substantial disease burden across all age groups and geographical regions [1]. The condition is defined by persistent inflammation of the mucosal lining of the nasal cavity and paranasal sinuses lasting more than 12 weeks, resulting in structural and functional changes within the sinonasal cavity, significant impairment of health-related quality of life, and considerable socioeconomic consequences [1,2]. The impact of the disease extends beyond the sinonasal tract, negatively affecting sleep quality, work productivity, social function, and general physical well-being, with associated costs attributable to repeated physician consultations, multiple antibiotic courses, and work absenteeism [3].

The American Academy of Otolaryngology - Head and Neck Surgery defines CRS as persistent inflammation of the nasal cavity and paranasal sinuses lasting longer than 12 weeks, characterized by both clinical symptoms and objective evidence of sinonasal disease on endoscopy or computed tomography imaging [2]. Major symptoms include nasal obstruction, facial pain or pressure, nasal discharge, hyposmia or anosmia, and post-nasal drainage, while minor symptoms include headache, halitosis, cough, dental pain, earache, and ear fullness [2]. The condition results from a complex interaction of inflammatory, infectious, anatomical, and immunological factors that impair mucociliary clearance and obstruct normal sinus drainage pathways, particularly within the osteomeatal complex [4]. Contributing factors include allergic rhinitis, nasal polyps, fungal infections, environmental pollutants, tobacco exposure, and host immune dysfunction [5,6].

Medical management with antibiotics, topical corticosteroids, antihistamines, and saline irrigation constitutes the first-line treatment for CRS; however, a significant proportion of patients fail to achieve adequate symptom control despite optimized medical therapy, necessitating surgical intervention [2,7]. Among available surgical approaches, functional endoscopic sinus surgery (FESS) has been established as the procedure of choice for medically refractory CRS, aiming to restore physiological sinus ventilation and drainage through targeted removal of obstructing pathological tissue [1,7].

Evidence from systematic reviews and meta-analyses consistently confirms that FESS is associated with significant improvement in symptom burden, patient-reported outcomes, and quality of life in appropriately selected patients [1,8]. Adjunctive pharmacological strategies, particularly intranasal corticosteroids and budesonide infiltration therapy, have shown promise in further reducing postoperative inflammatory activity and decreasing recurrence rates, especially in CRS with nasal polyps [4]. However, despite the established role of FESS in the management of medically refractory CRS, postoperative outcomes may vary across individual symptom domains, particularly with respect to olfactory recovery [5,6]. Furthermore, prospective studies evaluating symptom-specific outcomes in Indian patient populations remain relatively limited [2,3]. The present prospective analytical study was therefore conducted to provide local prospective evidence on short-term symptomatic outcomes following FESS, with particular emphasis on changes in individual CRS symptoms and the variability of postoperative olfactory improvement.

## Materials and methods

Study objectives

Primary Objective

The primary objective was to evaluate changes in the severity of key symptoms of CRS, including nasal obstruction, facial pain or pressure, post-nasal drip, and anosmia, six months after FESS in patients with medically refractory CRS.

Secondary Objectives

The secondary objectives were to assess the safety of FESS by documenting postoperative complications occurring during the follow-up period, compare the magnitude of improvement across individual symptom domains following surgery, and evaluate symptom-specific variability in postoperative outcomes, particularly the predictability of olfactory recovery (anosmia) compared with other CRS symptoms.

Study design

This was a prospective analytical cohort study designed to evaluate symptomatic improvement following FESS in patients with medically refractory CRS. The prospective approach allowed real-time data collection, minimizing recall bias and enabling systematic comparison of symptom severity before and after surgical intervention. The primary objective was to quantify symptomatic change using a standardized ordinal grading system and to determine the statistical significance of observed improvements using the Wilcoxon signed-rank test.

Study setting and duration

The study was conducted in the Department of Otorhinolaryngology of a tertiary healthcare institution operating as a referral center for patients with CRS, supported by dedicated endoscopic equipment, radiological facilities, and experienced surgical staff. The investigation was carried out between April 2025 and March 2026.

Study population and sampling

A total of 50 patients with CRS who fulfilled the eligibility criteria and provided written informed consent were enrolled in the study. All consecutive eligible patients presenting during the study period were recruited. Participants had previously received appropriate medical management, including antibiotics, topical nasal corticosteroids, antihistamines where indicated, saline irrigation, and decongestants, without achieving satisfactory symptom control. The sample size was considered adequate based on previous prospective studies evaluating symptomatic outcomes following FESS, which have reported clinically meaningful postoperative improvement using comparable patient cohorts [1]. Demographic and clinical characteristics, including age, sex, symptom duration, comorbidities, prior treatment history, postoperative symptom changes, and complications, were systematically recorded and analyzed.

Inclusion and exclusion criteria

Patients were required to have a diagnosis of CRS with symptoms persisting for more than 12 weeks, confirmed by clinical assessment, nasal endoscopy, and computed tomography of the paranasal sinuses. Both allergic and infective CRS subtypes were eligible. Patients were required to have failed a minimum six-week course of medical treatment, including antibiotics, topical nasal corticosteroids, antihistamines, saline irrigation, and decongestants as clinically indicated. Written informed consent for participation and FESS was mandatory.

Children under 12 years of age were excluded owing to anatomical and clinical differences in sinonasal disease between pediatric and adult populations. Patients with a prior history of nasal or sinus surgery were excluded, as were those with complex CRS requiring specialized surgical management and those with benign or malignant sinonasal lesions other than nasal polyps. These exclusions ensured a clinically homogeneous cohort of primary, uncomplicated CRS.

Symptom scoring and data collection

Symptom severity was assessed using a four-point ordinal symptom severity scale (0 = absent, 1 = mild, 2 = moderate, 3 = severe) adapted from previously published CRS outcome studies. Although this scale has been widely used for symptom-based assessment, formal psychometric validation was performed as part of the present study [2,3]. Postoperative outcomes were assessed using symptom severity scores obtained during follow-up visits. Objective postoperative endoscopic or radiological scoring systems were not routinely recorded and were therefore not included in the analysis. Additional qualitative symptom data, including the presence or absence of headache, halitosis, dental pain, cough, earache, and ear fullness, were recorded at each visit. Postoperative complications, including bleeding, synechiae formation, and major adverse events, were documented at each follow-up.

Ethics statement

This study was conducted in accordance with the principles of the Declaration of Helsinki. Ethical approval was obtained from the Institutional Review Board (IRB) of Narendra Modi Medical College, L.G. Hospital Campus, Maninagar, Ahmedabad, Gujarat, India (Reference Number: NaMoMC/IRB/2025/214; Approval Date: April 1, 2025). The study protocol, titled "Study on the effect of functional endoscopic sinus surgery on the symptom profile in patients with chronic rhinosinusitis," was reviewed and approved by the IRB at its meeting of March 28, 2025. Written informed consent was obtained from all study participants prior to enrollment.

Surgical procedure and postoperative care

All patients underwent FESS under general anesthesia following standard institutional protocols. The extent of surgery was individualized according to disease severity and preoperative computed tomography findings and included procedures such as uncinectomy, middle meatal antrostomy, anterior and/or posterior ethmoidectomy, sphenoidotomy, and frontal sinusotomy where indicated. The primary objective of surgery was restoration of physiological sinus ventilation and drainage while preserving normal mucosa as far as possible.

Procedures were performed by experienced otorhinolaryngology surgeons within the same tertiary care center using standard endoscopic techniques. Postoperatively, all patients received routine medical management, including saline nasal irrigation, intranasal corticosteroid therapy, and additional medications as clinically indicated. Patients were followed up at regular postoperative visits for endoscopic examination, assessment of healing, and identification of complications. Symptom assessment was performed preoperatively and at six months postoperatively using the predefined symptom severity scale. Outcome assessment was conducted by the treating clinical team and was not blinded.

Statistical analysis

All statistical analyses were performed using the Statistical Package for the Social Sciences ([SPSS], IBM Corp., Armonk, NY). Descriptive statistics were used to summarize demographic and clinical characteristics; continuous variables were expressed as mean ± standard deviation and median, and categorical variables were expressed as frequencies and percentages. The Wilcoxon signed-rank test was applied to compare paired preoperative and postoperative ordinal symptom scores, as this non-parametric test is appropriate for paired ordinal data that may not follow a normal distribution. Pairs with zero difference were excluded from the analysis using the standard Wilcoxon method. Effect size was estimated as r = Z/√N, where Z is the standardized test statistic and N is the number of non-zero difference pairs; r ≥ 0.5 was considered a large effect. Statistical significance was defined as p < 0.05.

## Results

Demographic characteristics

The study cohort comprised 50 patients with medically refractory CRS (Table 1). Of the 50 patients, 32 (64%) were male and 18 (36%) were female. The mean age was 37.66 ± 13.95 years (range 18-70 years). The most common age group was 21-30 years, accounting for 19 (38%) patients, followed by the 41- to 50-year group with 13 (26%) patients.

**Table 1:** Demographic characteristics of the study cohort (n = 50). SD, standard deviation

Characteristics	n	%
Gender (male)	32	64
Gender (Female)	18	36
Age group: <20 years	2	4
Age group: 21–30 years	19	38
Age group: 31–40 years	8	16
Age group: 41–50 years	13	26
Age group: 51–60 years	4	8
Age group: 61–70 years	4	8
Mean age ± SD (years)	37.66 ± 13.95	-
Median age (years)	35.5	-
Age range (years)	18–70	-

Preoperative symptom profile

All four assessed symptoms were common in the cohort (Table 2). Nasal obstruction was the most prevalent major symptom, present in 46 (92%) patients, with a mean preoperative severity score of 2.40 (median 3.0), indicating predominantly severe-to-moderate disease in this domain. Facial pain or pressure was present in 43 (86%) patients, with a mean preoperative score of 2.28 (median 3.0). Anosmia was present in 37 (74%) patients, with a mean score of 1.58 (median 2.0), while post-nasal drip was present in 34 (68%) patients, with a mean score of 1.54 (median 2.0). Qualitative assessment also identified the presence of headache (80%), halitosis (30%), ear fullness (30%), cough (10%), dental pain (10%), and earache (8%) in the cohort.

**Table 2:** Preoperative symptom severity scores among patients with chronic rhinosinusitis Scale: 0 = absent, 1 = mild, 2 = moderate, 3 = severe

Symptom	Patients with symptom (score > 0)	Prevalence (%)	Mean preoperative score	Median preoperative score
Nasal obstruction	46	92	2.4	3
Facial pain/pressure	43	86	2.28	3
Anosmia	37	74	1.58	2
Post-nasal drip	34	68	1.54	2

Postoperative symptom outcomes at six months

At six months following FESS, mean severity scores decreased for all four symptoms (Table 3). Nasal obstruction showed the greatest absolute reduction, with a mean score decline from 2.40 to 1.66. Of the 46 patients with preoperative nasal obstruction, 36 (78.3%) demonstrated score improvement; no patient showed worsening. Facial pain decreased from a mean of 2.28 to 1.54; 37 (86.0%) of 43 symptomatic patients showed improvement, with no cases of worsening. Post-nasal drip mean score declined from 1.54 to 1.24, with 15 (44.1%) of 34 symptomatic patients demonstrating score improvement; no patient worsened. Anosmia showed the smallest mean change, declining from 1.58 to 1.44. Of the 37 patients with preoperative anosmia, 11 (29.7%) showed improvement, 22 (59.5%) showed no change, and 4 (10.8%) demonstrated postoperative worsening of olfactory scores. These four patients who worsened had mild preoperative anosmia (score = 1) that progressed to moderate severity (score = 2) at six months.

**Table 3:** Comparison of preoperative and postoperative symptom severity scores at six months following functional endoscopic sinus surgery *Percentage of patients with preoperative symptom (score > 0) who demonstrated any reduction in score at six months.

Symptom	Mean preoperative score	Mean postoperative score	Mean reduction	Improved, n (%)*	Unchanged, n (%)	Worsened, n (%)
Nasal obstruction	2.4	1.66	0.74	36 (78.3)	10 (21.7)	0 (0.0)
Facial pain/pressure	2.28	1.54	0.74	37 (86.0)	6 (14.0)	0 (0.0)
Post-nasal drip	1.54	1.24	0.3	15 (44.1)	19 (55.9)	0 (0.0)
Anosmia	1.58	1.44	0.14	11 (29.7)	22 (59.5)	4 (10.8)

Analysis of preoperative and postoperative symptom severity scores

The Wilcoxon signed-rank test demonstrated statistically significant reductions in mean severity scores for three of the four symptoms (Table 4). Nasal obstruction showed the highest level of significance (W = 0.0, Z = 5.925, p < 0.001) with a near-perfect effect size (r = 0.987), indicating that every patient who showed any change improved. Facial pain similarly achieved extremely high statistical significance (W = 0.0, Z = 6.083, p < 0.001, r = 1.000), the largest Z-score in the dataset. Post-nasal drip reached statistical significance (W = 0.0, Z = 3.873, p < 0.001, r = 1.000), though the smaller number of responding patients reflects the greater proportion with no change at six months. Anosmia did not reach statistical significance (W = 32.0, Z = 1.807, p = 0.071, r = 0.467). The moderate effect size indicates that, among patients whose anosmia did change, both directions of change were represented. The presence of four patients with postoperative worsening of olfactory scores and the large proportion (59.5%) with no change at six months account for the non-significant result.

**Table 4:** Changes in symptom severity following functional endoscopic sinus surgery W statistic = Wilcoxon signed-rank test statistic; Z = standardized test statistic. The p-values were derived from the Wilcoxon signed-rank test comparing preoperative and six-month postoperative symptom severity scores. Effect size (r) was calculated as Z/√N, where N represents the number of non-zero difference pairs. Statistical significance was defined as p < 0.05. Effect sizes were interpreted as small (r = 0.10–0.29), moderate (r = 0.30–0.49), and large (r ≥ 0.50).

Symptom	W statistic	Z	p-value	Effect size (r)	Interpretation
Nasal obstruction	0	5.925	<0.001	0.987	Highly significant; large effect
Facial pain/pressure	0	6.083	<0.001	1	Highly significant; large effect
Post-nasal drip	0	3.873	<0.001	1	Significant; large effect
Anosmia	32	1.807	0.071	0.467	Not significant; moderate effect

Postoperative complications

FESS was associated with a low rate of minor complications. Postoperative bleeding occurred in four (8%) patients, all of whom were managed successfully with conservative measures without the need for further surgical intervention. Synechiae formation was identified in three (6%) patients at follow-up endoscopy and is amenable to outpatient endoscopic correction. No major complications, including cerebrospinal fluid leak, orbital injury, intracranial complications, or severe hemorrhage, were recorded in this series.

## Discussion

This prospective study demonstrated favorable symptomatic outcomes following FESS in patients with medically refractory CRS. Improvement was most evident in nasal obstruction and facial pain, whereas olfactory recovery remained variable. These findings support the established role of FESS in restoring sinonasal function while highlighting differences in postoperative response across symptom domains. The predominance of nasal obstruction and facial pain in this cohort is consistent with the typical clinical presentation of CRS reported in previous studies [2,9]. The marked postoperative improvement observed in these symptoms supports the effectiveness of FESS in restoring sinus ventilation and drainage, thereby reducing mucosal inflammation and symptom burden. These findings are in agreement with the systematic review by Algahtani et al. [1], which demonstrated consistent symptomatic benefit following FESS across diverse patient populations.

The findings regarding anosmia warrant careful interpretation. Although mean olfactory scores improved modestly following surgery, the change did not achieve statistical significance (p = 0.071), and four patients experienced worsening olfactory symptoms at six months. These findings suggest that olfactory recovery following FESS may be less predictable than improvement in other CRS symptoms. Olfactory dysfunction in CRS is often multifactorial and may result from both conductive impairment caused by mucosal edema, obstruction of the olfactory cleft, and nasal polyps, and sensorineural injury secondary to chronic inflammation of the olfactory neuroepithelium. Previous studies have identified baseline olfactory function, disease severity, duration of symptoms, and nasal polyp burden as important determinants of postoperative olfactory outcomes [5,10]. Patients with severe preoperative olfactory impairment or longstanding disease may have irreversible inflammatory or neuroepithelial changes that limit recovery despite technically successful surgery. Furthermore, recovery of olfactory function may continue beyond six months, and the follow-up period in the present study may not have been sufficient to capture delayed improvement occurring during prolonged postoperative healing and medical management [6]. These findings are consistent with the variable olfactory outcomes reported in the literature and highlight the complexity of restoring olfactory function in patients with CRS.

Post-nasal drip demonstrated a less pronounced response than nasal obstruction and facial pain. This may reflect the multifactorial nature of post-nasal drip, which can be influenced by allergic rhinitis, lower airway disease, and laryngopharyngeal reflux in addition to sinonasal inflammation. Previous studies have similarly reported that comorbid inflammatory conditions may limit complete symptom resolution following FESS [2,9]. Adjunctive therapies may further enhance surgical outcomes, as demonstrated by studies showing improved symptom control and lower recurrence rates when budesonide infiltration is combined with FESS [4] - a finding directly applicable to the patient population studied here. The complication profile in this series, limited to minor bleeding and synechiae with no major adverse events, is consistent with the established safety record of FESS reported in the literature. Dewey et al. in a comprehensive systematic review of systematic reviews confirmed that FESS consistently demonstrates very low rates of major complications in experienced centers [8].

The functional improvements observed in this study are consistent with previous reports demonstrating sustained postoperative improvement in quality-of-life measures following FESS in patients with CRS [3]. Similar benefits have also been reported in studies evaluating functional and symptomatic outcomes after endoscopic sinus surgery, further supporting the effectiveness of FESS in reducing disease burden and improving patient-reported outcomes [11].

Limitations

This study has several limitations that should be considered when interpreting the findings. First, the study was conducted at a single tertiary care center with a relatively small sample size, which may limit the generalizability of the results to broader patient populations. Second, the absence of a control group precludes direct comparison between surgical and non-surgical management and limits causal inference regarding treatment effects. Third, symptom assessment was based on a structured ordinal severity scale rather than a validated disease-specific quality-of-life instrument such as the Sino-Nasal Outcome Test-22 (SNOT-22). Finally, the six-month follow-up period may not fully capture long-term symptom control, disease recurrence, or the durability of surgical outcomes. Larger multicenter studies incorporating validated quality-of-life measures and longer follow-up are warranted to further define the long-term effectiveness of FESS in CRS.

## Conclusions

In this prospective cohort of patients with medically refractory CRS, FESS was associated with significant improvement in nasal obstruction, facial pain, and post-nasal drip at six months following surgery, while improvement in anosmia was less predictable. The procedure was associated with a low complication rate. However, interpretation of these findings should consider the study's single-center design, relatively small sample size, absence of a control group, use of a non-validated symptom severity scale, and limited follow-up duration. Further studies incorporating validated quality-of-life measures, objective outcome assessments, and longer follow-up are needed to better define long-term outcomes following FESS.
